# Segmental strain in chronic ischemic cardiomyopathy: clarifying the role of multiple-comparison adjustment and the clinical interpretation of limits of agreement

**DOI:** 10.1186/s44348-026-00076-7

**Published:** 2026-06-12

**Authors:** Maajid Mohi Ud Din Malik, Rajesh Prasad Jayaswal

**Affiliations:** 1 Department of Radiology and Imaging Technology, Dr. D.Y. Patil School of Allied Health Sciences, Dr. D.Y. Patil Vidyapeeth (Deemed to Be University), Pune, 411018 India; 2 Department of Medical Laboratory Technology, University Institute of Allied Health Sciences, Chandigarh University, Mohali, India

**Keywords:** Magnetic Resonance Imaging, Cine, Echocardiography, Ventricular Function, Left, Myocardial Ischemia, Data Interpretation, Statistical, Models, Statistical

## Abstract

Khidr and colleagues recently reported an intermodality comparison of left ventricular longitudinal strain by feature-tracking cardiac magnetic resonance (FT-CMR) and 2D speckle-tracking echocardiography (2D-STE) in 55 stable patients with chronic ischemic heart disease and reduced ejection fraction. Their analysis showed a strong global longitudinal strain (GLS) correlation (r = 0.793, P < 0.001) and explicitly cautioned against treating the two modalities as interchangeable for individual or longitudinal assessment. We support these conclusions and write to extend two methodological points. First, the 17 segment-level paired comparisons in Table 4 are reported without explicit multiplicity adjustment. On recomputation against a Bonferroni threshold of α/17≈0.00294, 11 of 17 segmental differences remain statistically significant, indicating that the original segmental signal is more statistically robust than an unadjusted reader might assume; we therefore recommend explicit reporting of adjusted P-values together with a unified mixed-effects formulation that accommodates within-patient segment-to-segment correlation. Second, the GLS limits of agreement span 8.3 percentage points (− 3.2% to + 5.1%), a width sufficient to misclassify individual patients across established GLS thresholds. A worked example is provided, and the recommendation to confine longitudinal follow-up to a single platform is argued to deserve more prominent placement in the Abstract and Conclusions.

Khidr et al. [1] examined intermodality concordance between feature-tracking cardiac magnetic resonance (FT-CMR) and 2D speckle-tracking echocardiography (2D-STE) for left ventricular longitudinal strain in patients with chronic ischemic cardiomyopathy and reduced systolic function. Pearson correlation was used to quantify linear association, and Bland–Altman analysis was used to characterize absolute agreement at both global and segmental levels. The global longitudinal strain (GLS) correlation (r = 0.793, P < 0.001) is consistent with prior reports in ischemic cohorts. The authors themselves are appropriately cautious in their Conclusions and state that the two methods cannot be considered interchangeable for individual GLS assessment, particularly where small changes in strain carry diagnostic or prognostic weight. We agree, and write to extend two specific points that, in our reading, deserve closer specification. The first concerns how the segment-level table is reported; the second concerns how the GLS limits of agreement (LoAs) are framed against clinical decision thresholds.

## Multiple-comparison handling in segmental analysis

Table 4 of the original article reports a Pearson coefficient and a paired-sample t-test for each of the 17 segments based on the standard American Heart Association model. No correction for multiplicity is described. Performing 17 simultaneous hypothesis tests at α = 0.05 yields a family-wise type I error rate of 1 − (1 − 0.05)^17^≈0.58. It is important to specify what this figure does and does not mean. Under the complete null hypothesis that no genuine intermodality difference exists at any segment, 0.58 is the probability of obtaining at least one false-positive P-value somewhere in the family; the expected count of false positives is 17 × 0.05≈0.85, not 5 or 6. We note this explicitly because the two quantities are easy to conflate, and an earlier formulation of the present concern risked doing so.

When the published P-values in Table 4 are checked against an adjusted threshold, the chance-finding concern is in fact constrained. Setting a Bonferroni cutoff at α/17≈0.00294, 11 of the 17 paired differences remain below this threshold: basal inferior, basal inferolateral, basal anterolateral, mid-inferoseptal, mid-inferior, mid-inferolateral, mid-anterolateral, apical septal, apical inferior, apical lateral, and the apex. A Benjamini–Hochberg false discovery rate procedure [2, 3] at q = 0.05 would retain a similar majority. The segmental signal of intermodality difference, in other words, is statistically supported where Khidr et al. [1] report it as strongest; a blanket dismissal of the segmental P-values as uninterpretable would overstate the matter. The substantive issue is one of reporting. The original article does not present adjusted P-values, and the reader is left to perform the correction. Explicit tabulation of family-wise or false discovery rate corrections, together with an indication of which segments retain significance under each scheme, would strengthen the segmental analysis without altering its direction.

Two segment-level patterns still warrant cautious reading even after adjustment. The basal anteroseptal segment yields P = 0.047 with a Pearson r of 0.028, an association effectively at zero; the paired t-test is driven here by a small fixed offset rather than by any measurement-tracking relationship between modalities. The mid-anterior segment shows a near-zero mean bias (0.049%) and yet LoAs of − 16.1% to + 16.1%. The absence of bias in this segment does not indicate agreement; it indicates symmetric scatter about the line of identity with no diagnostic information at the individual segment level. Both examples reinforce a more general point: paired-difference P-values and Bland–Altman widths describe different properties of measurement agreement, and segment-level conclusions are most secure when the two are read together.

## Within-patient clustering and unified inference

Each of the 17 paired t-tests in Table 4 is correctly grounded in 55 paired observations, one per patient for that segment. We do not suggest otherwise, and we wish to be clear on this point. The analytical design is less ideally specified at the level of inference across segments rather than within them. Adjacent myocardial segments in a single ventricle are mechanically coupled, share regional loading conditions, and in this cohort frequently share scar distribution along the left anterior descending territory, given that 100% of the included patients had a significant left anterior descending lesion. Segmental strain residuals from a single patient are therefore not independent of one another, and prior work has shown that regional strain values deteriorate in proportion to late gadolinium enhancement burden and transmurality within a given heart [4]. Running 17 separate paired comparisons does not exploit this within-patient structure and does not yield a single statistical statement about whether intermodality agreement varies meaningfully across segments after adjustment for it.

A more efficient and conceptually appropriate alternative is a linear mixed-effects model in which the intermodality difference is the outcome, segment is a categorical fixed effect (or, on the long-form dataset, segment × modality is specified as an interaction), and patient is a random intercept. Such a model accommodates the natural clustering of segmental observations within patients, produces a single global test for segment-dependent disagreement, and stabilizes segment-level estimates through partial pooling. We offer this as a methodological recommendation for future intermodality studies rather than as a verdict on the present one. The approach is now standard for clustered measurement-agreement designs and would have improved the inferential clarity of the segmental claims.

## Clinical interpretation of the GLS LoAs

Khidr et al. [1] report a mean bias of + 0.98% for FT-CMR minus 2D-STE and 95% LoAs from − 3.2% to + 5.1%, an agreement interval of 8.3 percentage points (their Fig. 5). The authors describe this as acceptable for global function estimation and themselves state, in the Conclusions, that the two methods cannot be considered interchangeable for individual GLS assessment when small changes in strain carry diagnostic or prognostic implications. We agree with that statement; our purpose here is to make its clinical implication more concrete than the present text allows.

Smiseth et al. [5] place the lower limit of normal GLS at approximately − 18%, with the − 14% to − 18% range corresponding to mild-to-moderate left ventricular dysfunction. An intermodality offset of approximately ± 4%, well within the reported LoAs, is sufficient to move a single patient with a true GLS near − 16% across the − 18% normal-abnormal boundary or, in the opposite direction, into the more clearly impaired range, depending only on which modality recorded the measurement on a given day. In settings where strain trajectories influence cardiotoxicity surveillance, viability assessment, or resynchronization eligibility, the risk of category-switching for a single patient is not abstract.

The agreement width reported by Khidr et al. [1] is comparable to that documented by the EACVI-ASE Strain Standardization Task Force across echocardiographic platforms [6]. This comparability underscores rather than relieves the issue: variability of this order appears to be intrinsic to current strain quantification, not peculiar to the present cohort. The natural corollary, which the original Discussion notes briefly, is that follow-up of a given patient should remain on a single platform and a single modality where the clinical question concerns small longitudinal change. We suggest this point belongs in the abstract and in the opening lines of the Conclusions, not toward the end, given its direct bearing on routine clinical use.

## Conclusions

Neither point raised here unsettles the central observation of Khidr et al. [1] that FT-CMR and 2D-STE track GLS similarly at the population level. The first point is one of statistical reporting: multiplicity adjustment, and in future work a mixed-effects formulation, would tighten the segmental inference without altering its substantive direction. The second is one of clinical communication: the agreement width observed in this cohort is broad enough to influence individual patient classification at established strain thresholds, and the recommendation to confine longitudinal follow-up to a single modality deserves more prominent placement than a single sentence in the Conclusions.

## Data Availability

No datasets were generated or analysed during the current study.

## Abbreviations

2D-STE: 2D speckle-tracking echocardiography
FT-CMR: Feature-tracking cardiac magnetic resonance
GLS: Global longitudinal strain
LoA: Limit of agreement
